# Digital Health Innovations in the Battle against COVID-19: A Global Perspective

**DOI:** 10.3390/healthcare11131892

**Published:** 2023-06-30

**Authors:** Yiannis Koumpouros

**Affiliations:** Department of Public and Community Health, University of West Attica, Athens 11596, Greece; ykoump@uniwa.gr

## 1. Introduction

The COVID-19 pandemic has unleashed unprecedented challenges upon the global population, demanding rapid and innovative solutions. Across various sectors, the incorporation of digital technologies, often prefixed with “tele-”, has played a pivotal role in reshaping societies and mitigating the impact of the virus [1,2]. This Special Issue focuses on the diverse range of digital solutions that have been proposed and adopted worldwide, showcasing the advancements in healthcare systems and the legislation that has influenced their adoption [3]. In response to the pandemic, countries around the world have implemented digital solutions to effectively manage and control the spread of the virus. Quarantine management systems, leveraging technologies such as geolocation and contact tracing apps, have proven invaluable in tracking and monitoring infected individuals. These tools have provided vital data for public health agencies, allowing for targeted interventions and resource allocation [4]. Identifying the sources and understanding the transmission dynamics of COVID-19 have been critical in developing effective containment strategies. Digital tools have facilitated the rapid sharing and analysis of vast amounts of genomic data, aiding scientists in tracking viral mutations and identifying potential variants of concern. Furthermore, monitoring infected populations and areas using intelligent information systems, sensor technologies, and IoT devices has enabled the early detection of outbreaks, leading to timely interventions and resource mobilization [5]. The pandemic has pushed healthcare systems to embrace digital transformation at an accelerated pace. Automation of in-hospital and out-of-hospital care processes has streamlined operations and reduced the risk of transmission, ensuring the safety of patients and healthcare professionals. Telecare and tele-rehabilitation have emerged as valuable alternatives to in-person consultations, enabling remote monitoring, personalized treatment plans, and continuity of care, especially for individuals with chronic conditions [6]. Digital solutions have played a crucial role in safeguarding the well-being of healthcare professionals and citizens alike. Robotics and AI technologies have been employed for tasks such as disinfection, the delivery of medical supplies, and even providing companionship to isolated patients. Furthermore, mHealth applications have empowered individuals to actively participate in monitoring their health, facilitating the early detection of symptoms and reducing unnecessary visits to healthcare facilities [7].

## 2. Special Issue Scope

This Special Issue presents valuable insights from nine articles in the field under investigation [3]. Specifically, González Aguña et al. [8] explore healthcare professionals’ perceptions of care robotics during the COVID-19 pandemic and their correlation with healthcare assessment tools. Schladen et al. [9] focus on developing and testing the HUGS system for monitoring infant neuromotor development, highlighting the challenges faced during the pandemic. Koutsouris et al. [10] propose a method for estimating the average time-to-recovery for contagious diseases, with a focus on COVID-19. Zafar et al. [11] investigate the impact of an enhanced preoperative evaluation process on bariatric surgery patients. Hsieh et al. [12] analyze factors influencing the continuance intention of using telehealth systems. Wu et al. [13] compare the perception of telemedicine between outpatients and medical staff. Al-Rayes et al. [14] assess the awareness and use of virtual clinics in Saudi Arabia. Camacho-Leon et al. [15] provide a narrative review of telemedicine adoption in Latin America during the pandemic. Lastly, Moussa et al. [16] examine the telehealth readiness of healthcare providers in Saudi Arabia, finding moderate to high levels of readiness, with financial contributions being a concern for nurses and physicians. Understanding healthcare providers’ readiness is crucial for successful telehealth implementation. While digital innovations have shown immense promise in the fight against COVID-19, challenges remain [17]. Objective and subjective assessments are necessary to evaluate the efficacy and usability of these digital solutions across different contexts and populations. Privacy and security concerns surrounding the collection and use of personal health data demand careful attention to maintain public trust [4,18]. Socioeconomic disparities and varying healthcare systems have influenced the adoption and effectiveness of digital solutions, necessitating a holistic approach to ensure equitable access and outcomes.

## 3. Conclusions

The COVID-19 pandemic has presented the world with an unprecedented opportunity to embrace digital health innovations [19]. The rapid advancements in sensor technologies, artificial intelligence, deep learning, and the IoT have catalyzed transformative changes in healthcare systems globally [20]. This Special Issue serves as a comprehensive exploration of the multifaceted digital solutions employed to combat the pandemic. By analyzing the objective and subjective assessments, addressing privacy concerns, and considering the socioeconomic impact, we can pave the way for a future where digital health technologies become an integral part of resilient and responsive healthcare systems worldwide.
